# Alantolactone inhibits cell autophagy and promotes apoptosis via AP2M1 in acute lymphoblastic leukemia

**DOI:** 10.1186/s12935-020-01537-9

**Published:** 2020-09-09

**Authors:** Ce Shi, Wenjia Lan, Zhenkun Wang, Dongguang Yang, Jia Wei, Zhiyu Liu, Yueqiu Teng, Mengmeng Gu, Tian Yuan, Fenglin Cao, Jin Zhou, Yang Li

**Affiliations:** 1 Central Laboratory of Hematology and Oncology, The First Affiliated Hospital, Harbin Medical University, Harbin, 150001 Heilongjiang China; 2 Department of Hematology, The First Affiliated Hospital, Harbin Medical University, Harbin, 150001 Heilongjiang China; 3 Department of Hematology, Tianjin Medical University Cancer Institute and Hospital, Tianjin, 300060 China

**Keywords:** Alantolactone, Acute lymphoblastic leukemia, Adaptor related protein complex 2 subunit mu 1, Autophagy, Apoptosis

## Abstract

**Background:**

Acute lymphoblastic leukemia (ALL) is an aggressive hematopoietic malignancy that is most commonly observed in children. Alantolactone (ALT) has been reported to exhibit anti-tumor activity in different types of cancer. The aim of the present study was to investigate the anti-tumor activity and molecular mechanism of ALT in ALL.

**Methods:**

ALL cell lines were treated with 1, 5 and 10 μM ALT, and cell viability was assessed using an MTT assay and RNA sequencing. Flow cytometry, JC-1 staining and immunofluorescence staining assays were used to measure cell apoptosis and autophagy. Additionally, western blot analysis was used to detect expression of apoptosis and autophagy related proteins. Finally, the effects of ALT on tumor growth were assessed in a BV173 xenograft nude mouse model.

**Results:**

ALT inhibited the proliferation of ALL cells in a dose-dependent manner. Additionally, it was demonstrated that ALT inhibited cell proliferation, colony formation, autophagy, induced apoptosis and reduced tumor growth in vivo through upregulating the expression of adaptor related protein complex 2 subunit mu 1 (AP2M1). Moreover, the autophagy activator rapamycin, attenuated the pro-apoptotic effects of ALT on BV173 and NALM6 cell lines. Overexpression of AP2M1 decreased the expression of Beclin1 and the LC3-II/LC3-1 ratio, and increased p62 expression. Knockdown of Beclin1 increased the levels of bax, cleaved caspase 3 and cytochrome C, and decreased bcl-2 expression.

**Conclusions:**

The present study demonstrated that ALT exerts anti-tumor activity through inducing apoptosis and inhibiting autophagy by upregulating AP2M1 in ALL, highlighting a potential therapeutic strategy for treatment of ALL.

## Background

Acute lymphoblastic leukemia (ALL) is the most common type of leukemia, and is characterized by uncontrolled proliferation of immature lymphoid cells [1, 2]. Despite significant advances over the past few decades to develop treatments for ALL, ~ 25% of children and half of the adults do not respond to chemotherapy or relapse [3–5]. At present, the available treatment options for ALL include chemotherapy, antibody therapy and allogeneic bone marrow transplantation, and the treatment used depends on the stage of cancer. Antibody therapy such as anti-CCR4, daclizumab and alemtuzumab, combined treatment with AZT and IFN, and allogeneic bone marrow transplantation have been suggested to cure ALL. Unfortunately, due to the various limitations, the ideal regimen has not yet been achieved [6].

ALT, a major bioactive sesquiterpene component of *Inula helenium*, has been reported to possess multiple biological and pharmacological properties including antibacterial, antifungal, anti-inflammatory and anticancer effects [7]. In recent years, ALT has been reported to exert anti-tumor effects in several types of cancer, including lung cancer, gastric cancer, hepatic cancer, B cell acute lymphoblastic leukemia, pancreatic cancer and breast cancer [8]. Moreover, ALT has been shown to exhibit synergistic anti-tumor effects with other therapeutics. For example, Wang et al. reported that ALT could enhance the sensitivity of lung cancer cells to gemcitabine [9]. Cao et al. stated that ALT may improve the therapeutic efficiency of the chemotherapeutic drug oxaliplatin [10]. Zheng et al. demonstrated that ALT could sensitize human pancreatic cancer cells to EGFR inhibitors [11]. The anti-tumor properties of ALT have been shown to occur through a range of molecular mechanisms. ALT promotes ROS-mediated inhibition of the Akt/glycogen synthase kinase (GSK)3β pathway and induces endoplasmic reticulum (ER) stress [9]. ALT regulates the p38 MAPK and NF-κB pathways. ALT inhibits TrxR1 activity and activates a ROS-mediated p38 MAPK signaling pathway [12]. Additionally, ALT impairs the autophagy-lysosome pathway by targeting TFEB [12]. Finally, ALT enhances the sensitivity of cancer cells to EGFR inhibitors through inhibition of STAT3 signaling [11]. These pathways are overlapping and interact with each other. However, the role and molecular mechanisms of ALT in ALL remain unexplored.

AP2M1 encodes the μ-subunit of the adaptor protein complex 2 (AP-2), which belongs to the adaptor complexes medium subunits family. AP2M1 was initially shown to be involved in clathrin-mediated endocytosis and intracellular trafficking. AP2M1 is one of the most important cytoplasmic carrier domains in clathrin-mediated endocytosis, and phosphorylation of this subunit stimulates clathrin and supports cell surface receptor incorporation. AP2M1 is required for hepatitis C, rabies and dengue virus infection, and AP2M1 knockdown reduces the viral titer or infectivity [13–15]. Moreover, abnormal AP2M1 causes developmental and epileptic encephalopathy through impaired clathrin-mediated endocytosis [16]. In addition, AP2M1 is involved in the transmission of secreted signals produced by senescent cells [17]. Recently, AP2M1 has been found to be associated with several types of cancer. Cong–Cong Wu et al. demonstrated that the expression of AP2M1 was significantly elevated in adenoid cystic carcinoma and mucoepidermoid carcinoma, and its expression was closely correlated with the expression of the proliferation marker, cyclin D1 [18]. AP2M1 can also serve as a prognostic marker of hepatocellular carcinoma. AP2M1, as a transcription factor, specifically binds to the hepatocyte growth factor gene promoter to repress its activity [19, 20]. Together, these previous studies highlight the diverse roles of AP2M1 in several human diseases.

In this study, we first demonstrated that ALT inhibited the proliferation of ALL cells in a dose-dependent manner. Importantly, AP2M1 was shown to mediate the anti-tumor effects of ALT on BV173 and NALM6 cells. Subsequently, the relationship between autophagy and apoptosis was evaluated by administration of rapamycin. Finally, the effects of overexpressing AP2M1 on autophagy and the effect of Beclin1 knockdown on apoptosis were examined.

## Methods and materials

### Cell culture and reagents

The human ALL cell lines (BV173, NALM6, JM-1, NALM1, RS6 and SUPB15) were purchased from the Cell Bank of the Chinese Academy of Sciences (Shanghai, China). All cell lines were cultured in DMEM supplemented with 10% FBS (Hyclone; Cytiva) at 37 °C with 5% CO_2_ in a humidified incubator. ALT and rapamycin were obtained from MedChemExpress (New Jersey, USA). Both the tested compounds were dissolved in DMSO. The final concentration of DMSO in culture medium did not exceed 0.1%.

### Cell viability assay

Cell viability was assessed using a MTT assay kit (Sigma-Aldrich; Merck KGaA). BV173 and NALM6 cells were seeded in 96-well plates and then treated with 1, 5 and 10 μM of ALT for 24 h. Subsequently, 10 μl 5 mg/ml MTT solution was added, and incubated at 37 °C for 2 h. MTT solvent was then added to the culture. Absorbance was measured at 570 nm using a microplate reader. Each experiment was performed at least three times.

### RNA sequencing

All of the RNA-sequencing procedures were performed by the Beijing Genomics Institute (BGI, Shenzhen, China). Briefly, BV173 and NALM6 cells were treated with ALT (5 μM) and control (0.1% DMSO) for 24 h. Total mRNA was isolated using TRIzol reagent (Invitrogen; Thermo Fisher Scientific, Inc.), and reversed transcribed into a library of cDNA fragments using TruSeq Stranded mRNA LTSample Prep Kit (Illumina, Inc., San Diego, USA). Subsequently, these samples were sequenced on the Illumina HiSeq 4000 platform (Illumina, Inc. San Diego, USA). Statistical analysis was performed using the ALEXA-seq software package, and differentially expressed genes (DEGs) were selected based on a selection criteria of a fold change ≥ 2, P < 0.05 and false discovery rate < 0.05.

### Plasmids transfection

The small interfering (si) RNA for AP2M1 (si-AP2M1), Beclin1 (si-Beclin1), pcDNA3.1 vector AP2M1 overexpression vector and negative controls were obtained from Shanghai GenePharma Co., Ltd. (Shanghai, China). The sequences of siRNA were: si-AP2M1, GGGUGGUGAUGAAGAGCUACC and si-Beclin1, GGUGUUUGAUACUGUUUGAGA. Plasmids were transfected into ALL cells using Lipofectamine 2000 (Invitrogen; Thermo Fisher Scientific, Inc.) according to the manufacturer’s protocol.

### Colony formation assay

Cells were seeded in 12-well plates (500 cells/well) and incubated for 14 days and then fixed with 4% paraformaldehyde for 15 min. The fixed cells were stained with 0.1% crystal violet, and then imaged using a microscope. The number of visible cell colonies were recorded.

### Cell apoptosis

Following treatment with different concentrations of ALT, both BV173 and NALM6 cells were harvested and washed with PBS 3 times. For cell apoptosis analysis, cells were labeled with Annexin V-Fluorescein Isothiocyanate (FITC)/propidium iodide (PI) according to manufacturer’s protocol. Finally, cells were observed and imaged using a BD FACSCalibur flow cytometry system (Becton–Dickinson, NJ, USA).

### JC-1 staining

JC-1 staining was used to assess the mitochondrial membrane potential (MMP) using a JC-1 assay kit (Beyotime Institute of Biotechnology, Shanghai, China) according to the manufacturer’s protocol. Images were obtained using a fluorescence microscope (Leica, Wetzlar, Germany). The ratio (%) of red/green fluorescence intensity was calculated using ImageJ version 2.1 (National Institutes of Health, Bethesda, MD, USA).

### Western blot analysis

Anti-AP2M1, anti-cytochrome C and anti-cleaved caspase-3 antibodies were obtained from Abcam (Shanghai, China). Anti-Beclin 1, -p62, -LC3, bcl-2, -bax, -cleaved-caspase3 and GAPDH antibodies were obtained from ProteinTech (Shanghai, China). Total proteins were extracted using RIPA-lysis buffer supplemented with 10 mM PMSF (Beyotime Institute of Biotechnology, Shanghai, China). A bicinchoninic acid assay was used to quantify protein concentrations. A total of 40 μg protein was loaded on a 10% SDS-gel, resolved using SDS-PAGE and transferred to polyvinylidene fluoride membranes. Then, the membranes were blocked with 5% nonfat milk in Tris-buffered saline and 0.1% Tween 20 (TBST), and incubated with primary antibodies at 4 °C overnight. The following day, the membranes were washed 5 times with TBST, the membranes were then incubated with secondary antibodies for 2 h. An enhanced chemiluminescence system kit (Beyotime Institute of Biotechnology, Shanghai, China) was used to visualize the signals. Densitometry analysis was performed using ImageJ.

### Immunofluorescence

BV173 and NALM6 cells were re-suspended to prepare cell suspensions following treatment with the designated reagents. A total of 40 µl cell suspension was dropped on coverslips. Subsequently, the coverslips covered with cells were dried in an oven at 50 °C, and then washed with PBS. Next, cell coverslips were fixed in 4% paraformaldehyde for 15 min, and permeabilized with 0.2% Triton X-100 for 15 min. Subsequently, the cells were sealed with 5% BSA solution at 37 °C for 40 min, and then incubated with anti-AP2M1 primary antibodies at 37 °C for 4 h. The cells were incubated with the Alexa Fluor^®^-594 or 488 secondary antibodies (Abcam, Shanghai, China) at 37 °C for 1 h and stained with DAPI for 3 min. Finally, antifade mounting reagent was placed on the slide and covered with a cell-coated glass sheet. The glass slides were observed using a fluorescence microscope (Leica, Wetzlar, Germany). ImageJ was used to measure the fluorescence intensity.

### Confocal analysis

For confocal microscopy, the GFP-LC3 vector was transfected into the BV173 cells to generate a stable GFP-LC3 BV173 cell line. Following treatment, the GFP-LC3 BV173 cells were fixed in 4% paraformaldehyde for 15 min. Subsequently, the cells were permeabilized using 0.05% Triton X-100 and stained with DAPI (Invitrogen; Thermo Fisher Scientific, Inc., Waltham, MA, USA). Cells were examined and quantified using an AOBS confocal laser scanning (Leica, Wetzlar, Germany).

### Xenograft model

Experiments were performed in BALB/c nu/nu mice (6 weeks old), which were obtained from Charles River Laboratories, Inc. Wilmington, MA, USA. A total of 3x10^6^ BV173 cells transfected with AP2M1 siRNA in 100 μl PBS were subcutaneously injected into the posterior flank region of nude mice. The long diameter and short diameter of tumors were measured every 2 days, and the volume was calculated as follows: Tumor volume = 0.5× long diameter x short diameter^2^. The mice were treated with ALT every day after injection for 15 days. Finally, the mice were sacrificed and the tumors were excised.

### Statistical analysis

SPSS version 22.0 (IBM, Corp.) was used to analyze the data. Data are presented as the mean ± standard deviation. A one-way ANOVA followed by a Tukey’s post hoc test was used to assess the difference between multiple groups. Differences between two groups were analyzed using a Student’s t-test. P < 0.05 was considered to indicate a statistically significant difference.

## Results

### ALT inhibits the proliferation of ALL cells

**Fig. 1:**
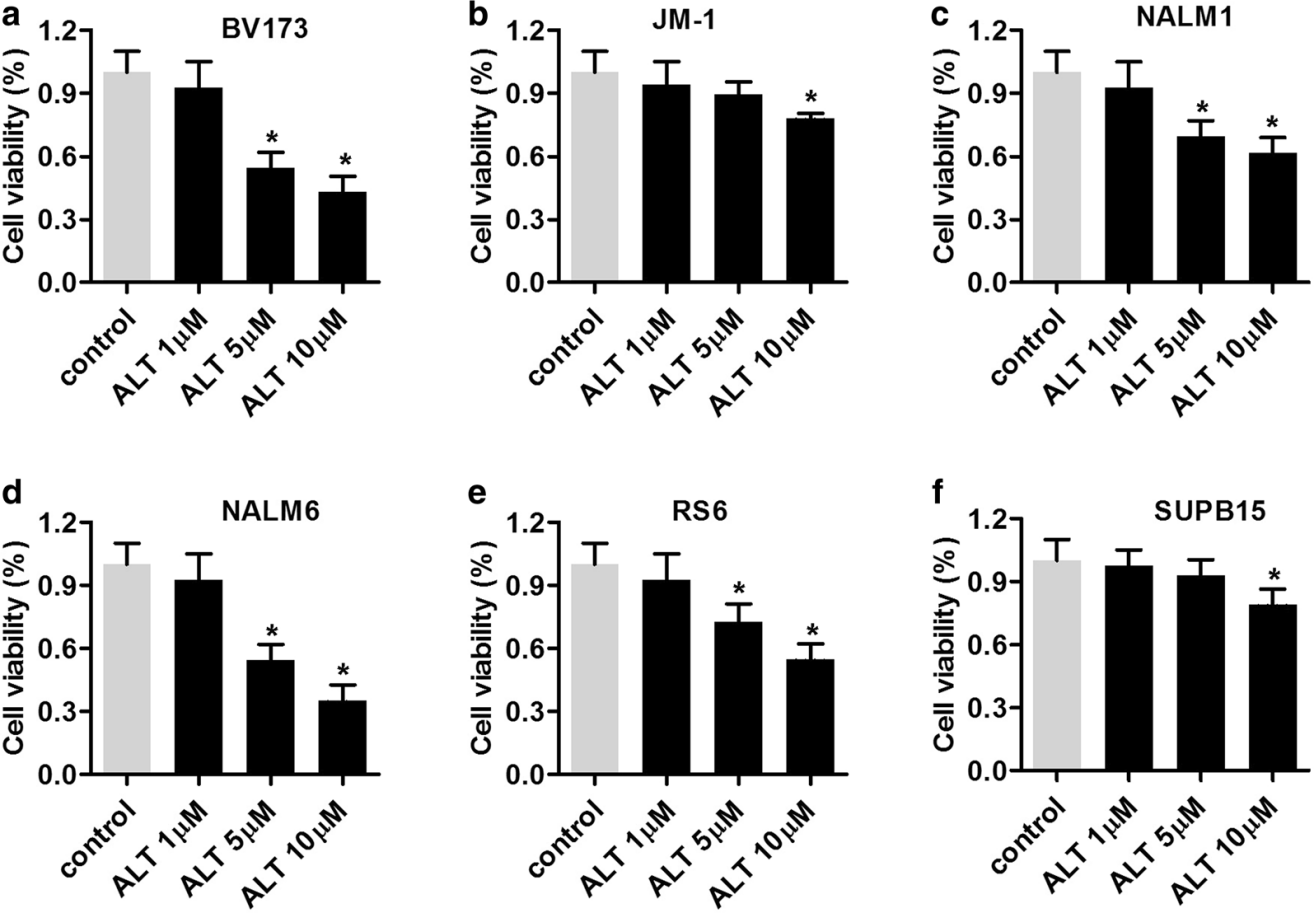
ALT inhibits the proliferation of ALL cells. ALL cell lines (**a**) BV173, (**b**) JM-1, (**c**) NALM1, (**d**) NALM6, (**e**) RS6 and (**f**) SUPB15 were treated with 1, 5 and 10 μM ALT for 24 h and cell viability was assessed. Data are presented as the mean ± standard deviation of three repeats. ^*^P < 0.05, ^**^P < 0.01 vs. control. ALL, acute lymphoblastic leukemia; ALT, alantolactone

To investigate the potential effects of ALT on ALL, ALL cell lines, including BV173, JM-1, NALM1, NALM6, RS6, and SUPB1, were treated with 1, 5 and 10 μM of ALT for 24 h. Cell viability was determined using an MTT assay. The results showed that both 5 and 10 μM ALT significantly inhibited the proliferation of BV173 (Fig. 1a), NALM1 (Fig. 1d), NALM6 (Fig. 1c), and RS6 cells (Fig. 1e), and 10 μM ALT inhibited the proliferation of JM-1 (Fig. 1b) and SUPB1 cells (Fig. 1f). Additionally, 5 μM ALT significantly reduced growth of BV173 and NALM6 cell lines, and thus, these cell lines were used for subsequent experiments.

### ALT treatment increases expression of AP2M1

**Fig. 2:**
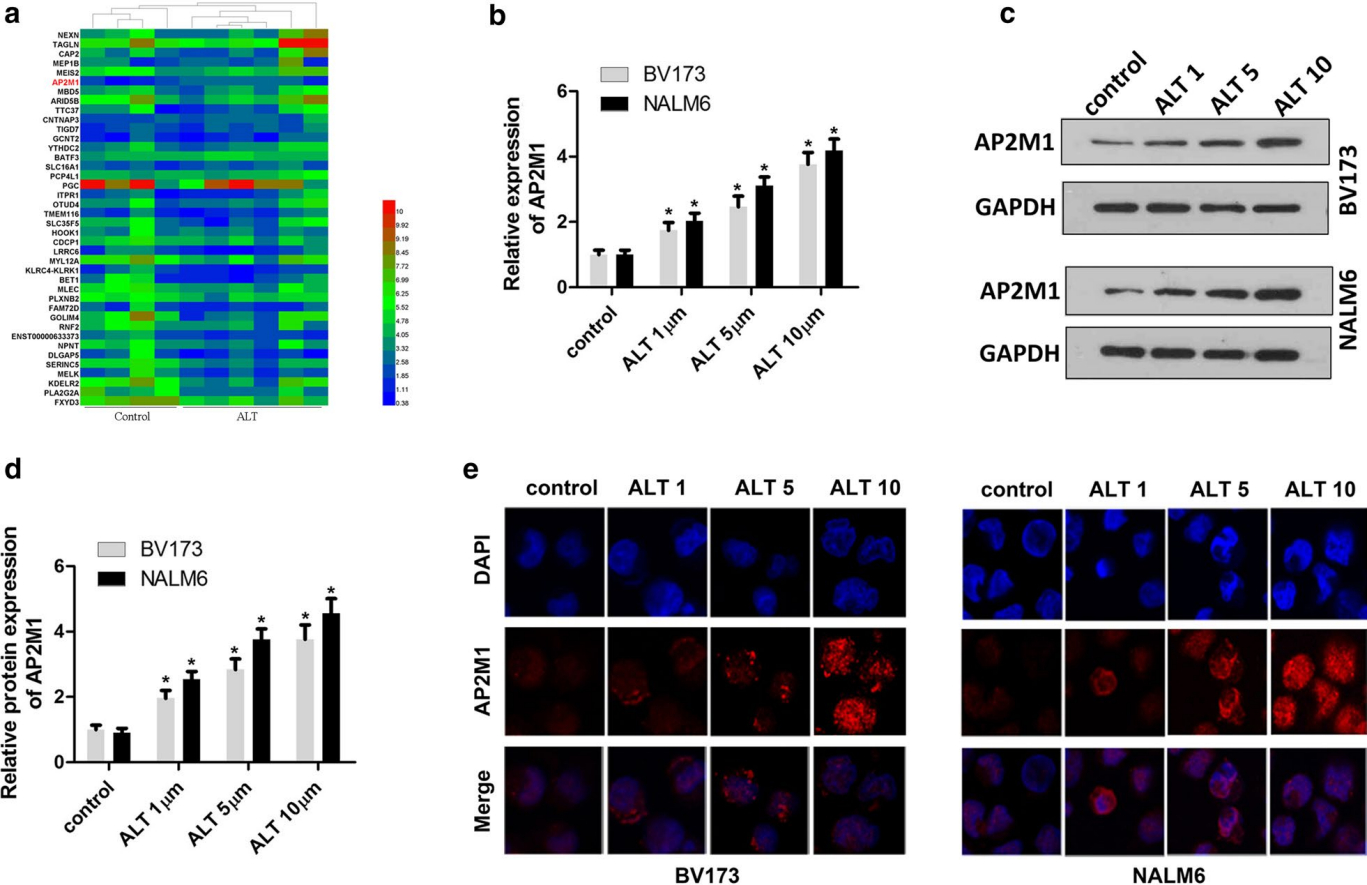
ALT promotes the expression of AP2M1. **a** RNA-sequencing was performed to screen the differentially expressed genes in ALL cells treated with ALT. Following treatment with different concentrations of ALT, **b** qPCR, (**c**, **d**) western blotting, and (**e**) immunofluorescence analysis were performed to evaluate the mRNA and protein expression levels of AP2M1 in BV173 and NALM6 cells. ^*^P < 0.01 vs. control. ALL, acute lymphoblastic leukemia; ALT, alantolactone; AP2M1, adaptor related protein complex 2 subunit mu 1

To elucidate the mechanism underlying the effects of ALT on ALL cells, we screened the mRNA expression profiles of cells using RNA-seq. As shown by the heatmap (Fig. 2a), AP2M1 expression was notably upregulated in both BV173 and NALM6 cells treated with 5 μM ALT. To confirm this result, RT-qPCR and western blotting were performed to detect AP2M1 expression in BV173 and NALM6 cells. As expected, a dose-dependent increase of AP2M1 mRNA and protein expression was observed in ALT-exposed BV173 and NALM6 cells (Fig. 2b–d). Furthermore, cell immunofluorescence assay visually demonstrated that ALT promoted an increase in AP2M1 protein expression levels in a dose-dependent manner (Fig. 2e). Accordingly, these results indicate that ALT treatment induces the expression of AP2M1 in ALL cell lines.

### ALT inhibits proliferation and colony formation of ALL cells by targeting AP2M1

**Fig. 3:**
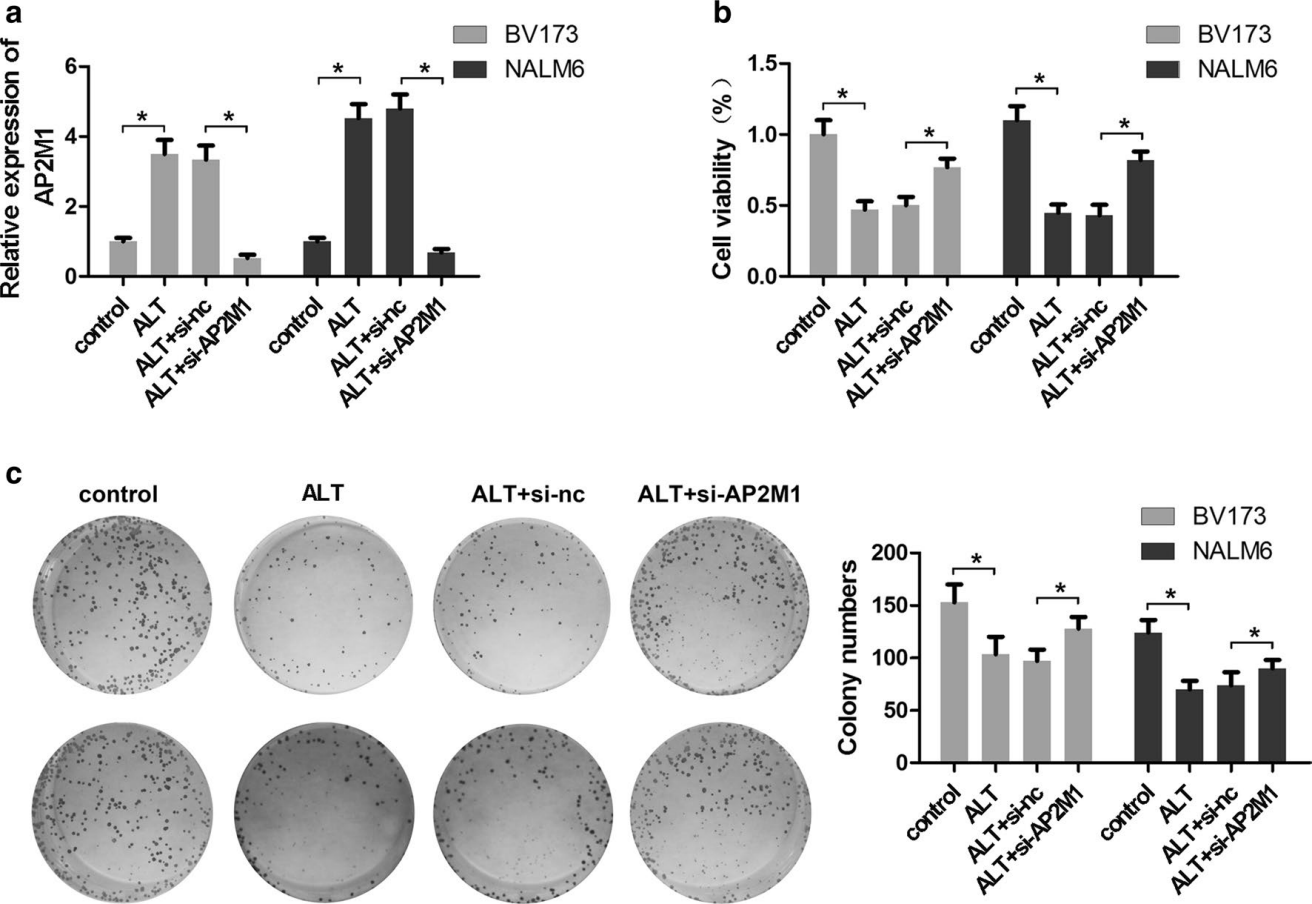
AP2M1 knockdown reverses the inhibitory effects of ALT on ALL cell proliferation. BV173 and NALM6 cells were transfected with si-AP2M1 or si-NC, and then treated with 5 μM of ALT for 24 h. **a** qPCR was performed to evaluate the expression of AP2M1. **b** MTT assays were used to assess cell proliferation. **c** Colony formation assays were performed to investigate the colony formation ability of ALL cells. ^*^P < 0.05. siRNA, small interfering RNA; AP2M1, adaptor related protein complex 2 subunit mu 1; ALL, acute lymphoblastic leukemia; ALT, alantolactone

To determine whether AP2M1 is involved in the cytotoxicity of ALT, BV173 and NALM6 cells were co-treated with ALT and si-AP2M1. As shown in Fig. 3a, ALT upregulated AP2M1 expression while si-AP2M1 transfection significantly reduced AP2M1 expression, indicating AP2M1 siRNA could effectively inhibit the expression of AP2M1 in BV173 and NALM6 cells. Subsequently, the effect of ALT and si-AP2M1 on cell proliferation was assessed using MTT and colony formation assays. The MTT assay showed that ALT reduced cell viability of cells, while AP2M1 knockdown reversed the toxic effects of ALT, indicating that AP2M1 was involved in the toxic effects of ALT on cells (Fig. 3b). Consistent with the results from the MTT assays, the colony formation assay also showed the same conclusion (Fig. 3c). Collectively, the data demonstrate that ALT exerts a growth inhibitory effect on ALL cells via upregulation of AP2M1.

### ALT inhibits ALL growth in vivo

**Fig. 4:**
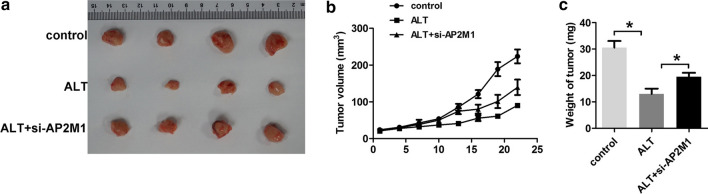
ALT inhibits the growth of acute lymphoblastic leukemia in vivo. BV173 cells transfected with AP2M1 siRNA in 100 μl PBS were subcutaneously injected into the posterior flank region of nude mice. After 15 days, mice were treated with ALT once daily. **a** Images of xenograft tumors dissected from each mouse. **b** Tumor volumes are indicated by the curves representing the trend in the increase in tumor size. **c** Mean weight of tumors. ^*^P < 0.05. ALT, alantolactone; AP2M1, adaptor related protein complex 2 subunit mu 1

A xenograft model experiment was used to further investigate the effects of ALT on ALL. As shown in Fig. 4b, from day 14, tumor volume in the ALT group was significantly lower compared with the control group, whereas xenograft tumor volume in the co-treatment group with ALT and si-AP2M1 was larger compared with the ALT treated group (alone) (P < 0.05). The xenograft tumors were weighed after the mice were sacrificed. As shown in Fig. 4a and c, tumor weight showed the same results. Accordingly, these results indicate ALT suppresses tumor growth in vivo, and this effect could be reversed by knockdown of AP2M1.

### ALT induces apoptosis and inhibits autophagy of ALL cells by upregulating expression of AP2M1

**Fig. 5:**
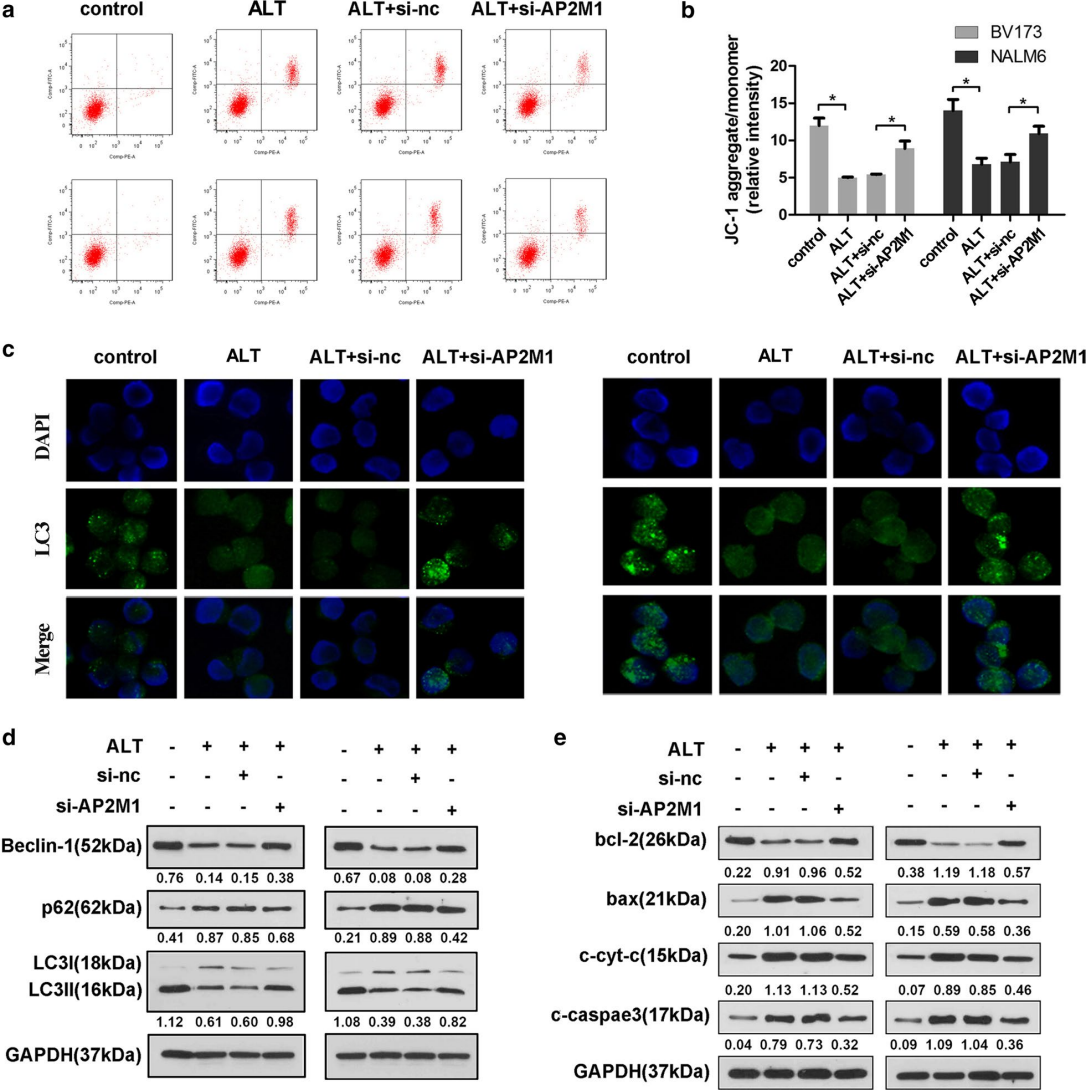
ALT induces apoptosis and inhibits autophagy of ALL cells. BV173 and NALM6 cells were transfected with si-AP2M1 or si-NC, and then treated with 5 μM ALT for 24 h. **a** Annexin/V-PI staining and flow cytometry were performed to assess cell apoptosis of ALL cells. **b** JC-1 staining was used to detect the mitochondrial membrane potential of the ALL cells. **c** LC3 fluorescence dots labeled by GFP were observed under the fluorescence microscope. **d** Western blotting was used to assess the expression of proteins. ^*^P < 0.05. ALL, acute lymphoblastic leukemia; ALT, alantolactone; si, small interfering; NC, negative control; AP2M1, adaptor related protein complex 2 subunit mu 1

The MMP is an important indicator of cell function and health, and its dissipation is considered an early indicator of cell apoptosis. To evaluate the effect of ALT on cell apoptosis, flow cytometry, JC-1 staining and western blotting were used to measure cell apoptotic rate, MMP and apoptosis-related proteins, respectively. As shown in Fig. 5a, treatment with 5 μM ALT for 24 h significantly increased the apoptotic rate of BV173 and NALM6 cells, whereas AP2M1 knockdown significantly reduced this pro-apoptotic effect of ALT. Meanwhile, JC-1 staining showed that ALT significantly decreased MMP of ALL cells, which was reversed by AP2M1 knockdown (Fig. 5b). Furthermore, western blotting showed that ALT treatment notably increased the levels of cleaved caspase-3, bax and cyt-c in the cytoplasm, and decreased bcl-2 expression, but these effects were attenuated by si-AP2M1 (Fig. 5e). These results suggest that knockdown of AP2M1 attenuates the pro-apoptotic effects of ALT by maintaining mitochondrial function and inhibiting the caspase cascade.

Next, we detected the autophagy of BV173 and NALM6 cells using immunofluorescence staining and western blotting. Immunofluorescence assay results showed that ALT treatment reduced the number of the LC3 fluorescent puncta compared with the control group, whereas AP2M1 knockdown increased the number of LC3 puncta compared with the ALT group (Fig. 5c). Additionally, western blotting analysis demonstrated that ALT significantly decreased the expression of autophagy markers Beclin1 and LC3II/LC3I ratio, and increased the expression of autophagy substrate p62 protein. However, ALT-mediated regulation of these autophagy-related proteins was reversed by AP2M1 knockdown, indicating the involvement of AP2M1 in ALT-mediated autophagy regulation (Fig. 5d). Together, these data support the notion that ALT induced apoptosis and inhibited autophagy of ALL cells via upregulation of AP2M1.

### Rapamycin-induced autophagy attenuates the pro-apoptotic effect of ALT

**Fig. 6:**
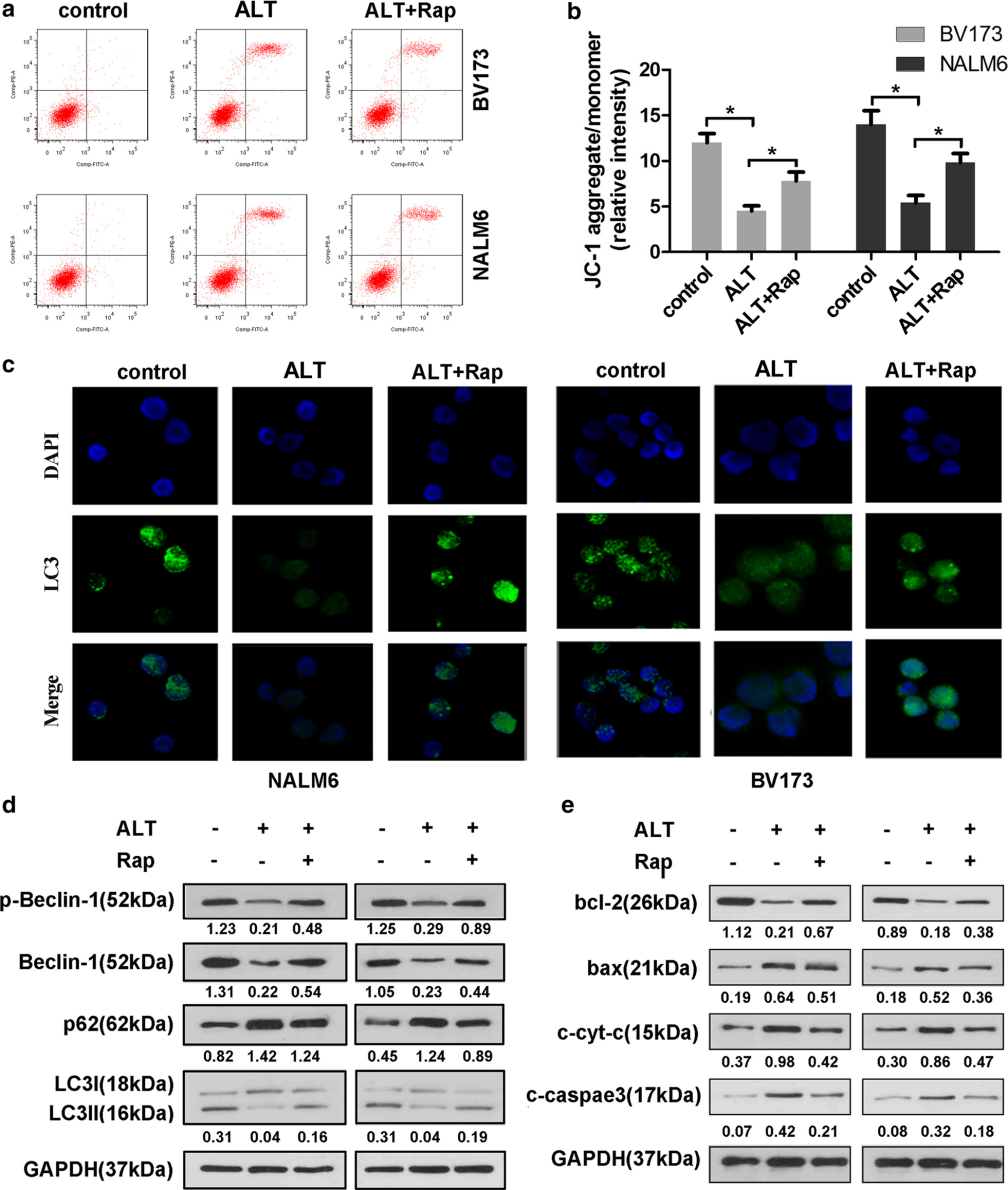
Induction of autophagy reverses the pro-apoptotic effects of ALT on BV173 and NALM6 cells. ALL cells were treated with ALT either alone or combined with rapamycin for 24 h. **a** Annexin/V-PI staining and flow cytometry were performed to detect cell apoptosis of ALL cells. **b** JC-1 staining was performed to detect the mitochondrial membrane potential of the ALL cells. **c** LC3 fluorescence dots labeled by GFP were observed using fluorescence microscopy. **d** Western blotting was used to investigate the expression of autophagy-related proteins. **e** Western blotting was used to investigate the expression of apoptosis-related proteins. ^*^P < 0.05. ALL, acute lymphoblastic leukemia; ALT, alantolactone

Autophagy and apoptosis are necessary to maintain cellular homeostasis. There is a complex relationship between autophagy and apoptosis. In general, autophagy signaling prevents the induction of apoptosis, and apoptosis-related caspase activation, as a feedback response, can inhibit the autophagic process. However, autophagy can also trigger apoptosis in certain cases. To further explore the relationship between autophagy and apoptosis induced by ALT, BV173 and NALM6 cells were treated with ALT alone or in combination with the autophagy activator, rapamycin. Immunofluorescence analysis results showed that ALT treatment reduced the number of LC3 fluorescent puncta compared with the control group, whereas rapamycin increased the number of LC3 puncta compared with the ALT group (Fig. 6c). Western blot analysis showed that rapamycin reversed the downregulation of Beclin1 and LC3II/LC3I ratio, and elevated p62 levels induced by ALT in BV173 and NALM6 cells (Fig. 6d), suggesting that rapamycin could efficiently activate autophagy. Furthermore, the effect of rapamycin on the pro-apoptotic activity of ALT was evaluated. The results showed that rapamycin partially abolished the increase in apoptotic rate and MMP caused by ALT, as demonstrated in Fig. 6a, b. Moreover, this finding was confirmed by assessing the expression levels of apoptosis related-proteins (Fig. 6e). These findings indicate that ALT could induce cell apoptosis via inhibition of autophagy in ALL.

### AP2M1 inhibits autophagy and induces apoptosis

**Fig. 7:**
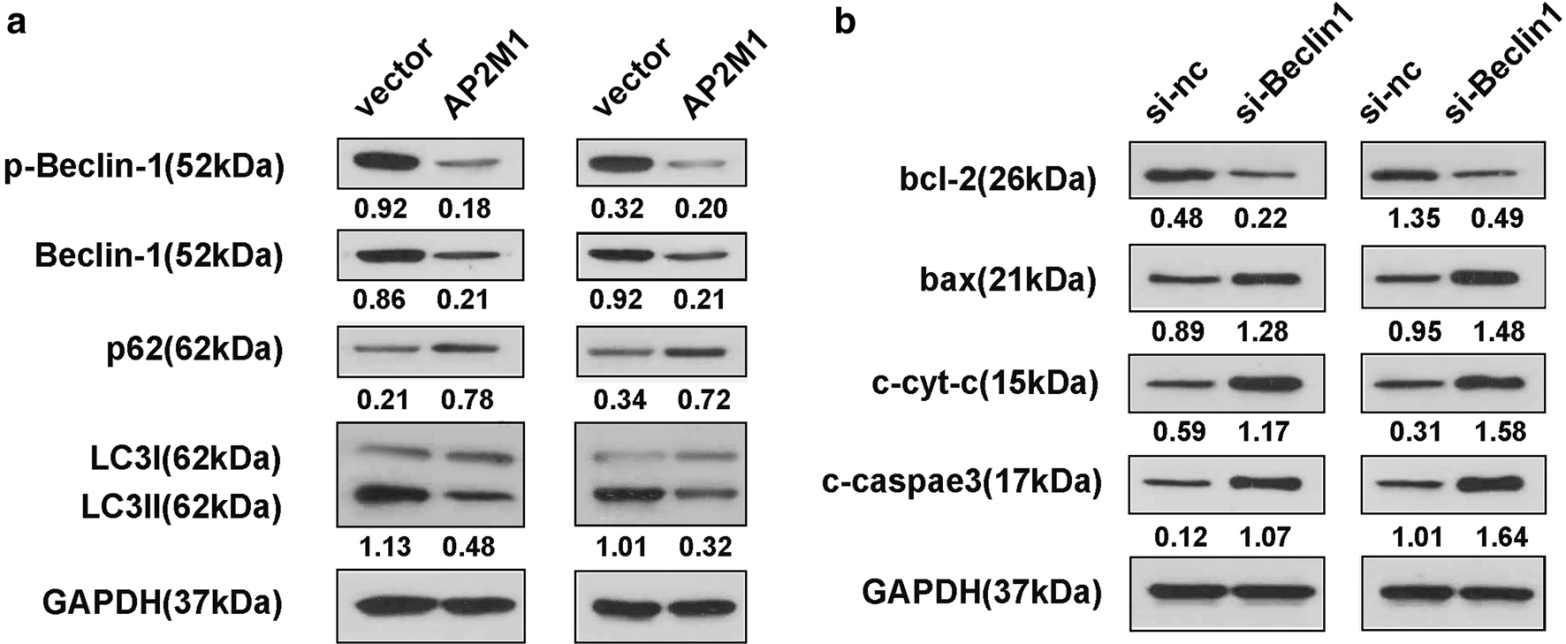
AP2M1 inhibits autophagy and induces apoptosis. ALL cells were transfected with pcDNA3.1-AP2M1 or si-Beclin1 for 48 h. Then, western blotting was used to evaluate the levels of (**a**) autophagy and (**b**) apoptosis-related proteins. AP2M1, adaptor related protein complex 2 subunit mu 1; ALL, acute lymphoblastic leukemia; si, small interfering

As AP2M1 is essential for ALT-mediated apoptosis and autophagy of ALL cells, we next examined whether overexpression of AP2M1 affects apoptosis and autophagy. Western blotting demonstrated that overexpression of AP2M1 inhibited the expression of the autophagy-related markers, Beclin1 and LC3II, and increased the expression of p62 (Fig. 7a), confirming inhibition of autophagy by AP2M1 in ALL cells. Moreover, the knockdown of Beclin1 caused a significant increase in the expression of apoptosis-related markers, such as bax, c-cyt-c and cleaved caspase 3 and a notable reduction in bcl-2 expression, indicating the crosstalk between autophagy and apoptosis. Taken together, the results of the present study suggest that ALT may maintain cellular homeostasis between autophagy and apoptosis by increasing AP2M1, and thereby exerting anti-tumor activity in ALL.

## Discussion

In the present study, ALT was found to promote apoptosis of ALL cells by inhibiting autophagy. In total, two novel findings are presented: First, this study provided evidence that ALT exerted its anti-tumor activity via regulation of autophagy and apoptosis in ALL cells. Additionally, it was shown that AP2M1 protein contributed to the anti-tumor effects of ALT on cancer cells by modulating autophagy.

There are studies which have shown that ALT exerts anti-tumor effects on tumor cells alone or when combined with other anti-tumor agents The mechanisms involved included regulation of oxygen species-mediated ER stress, ROS response, and modulation of other signaling pathways, such as the Akt/GSK3β, p38 MAPK, NF-κB, STAT3 and Nrf2 signaling pathways. Currently, the function of ALT on autophagy has only been reported once by He et al. who found that ALT caused the accumulation of autophagosomes due to impaired autophagic degradation, and significantly inhibited the activity and expression of CTSB/CTSD proteins [12]. Their data demonstrated that ALT, which impaired autophagic degradation, was a pharmacological inhibitor of autophagy in pancreatic cancer cells and markedly enhanced the chemosensitivity of pancreatic cancer cells to oxaliplatin. In the present study, we demonstrated that inhibition of autophagy was also the mechanism which underlies ALT-mediated suppression of the growth of ALL, and this result contributed novel evidence of the involvement of autophagy on the anti-tumor effects of ALT.

ALT has been shown to contribute to cell apoptosis in several types of cancer. For example, ALT induces gastric cancer BGC-823 cell apoptosis by regulating the AKT signaling pathway [21]. ALT induces apoptosis of breast cancer cells via the p38 MAPK, NF-κB and Nrf2 signaling pathways [22]. ALT also induces apoptosis and enhances the chemosensitivity of A549 lung adenocarcinoma cells to doxorubicin [23]. In the present study, it was shown that ALT also promoted the apoptosis of ALL cells.

Moreover, as suggested in the present study, ALT significantly stimulated apoptosis but at the same time inhibited autophagy of BV173 and NALM6 cells. This function was further confirmed by the use of rapamycin. The results showed that rapamycin significantly induced autophagy and reversed the effects of ALT on apoptosis, suggesting that ALT induces apoptosis partially through inhibition of autophagy. Necrosis, apoptosis and autophagy are three types of programmed cell death that are crucially involved in cancer cell progression, division and metastasis [23, 24]. However, the relationship between autophagy and apoptosis remains incompletely understood. In some cases, autophagy promotes cell apoptosis. Paris saponin-induced autophagy promotes ALL cell apoptosis through the Akt/mTOR signaling pathway [25]. Parthenolide inhibits pancreatic cell progression by autophagy-mediated apoptosis [26]. In other cases, autophagy inhibited the apoptotic process. For example, Rottlerin-stimulated autophagy results in apoptosis in bladder cancer cells [27]. Autophagy inhibition improves heat-stimulated apoptosis in human non-small cell lung cancer cells through ER stress pathways [28].

In order to determine the mechanisms underlying the effects of ALT on apoptosis and autophagy, we tried to identify the key factors involved in both apoptosis and autophagy. In the present study, AP2M1 was found to be upregulated in ALL cells treated with ALT. Furthermore, AP2M1 knockdown significantly inhibited the effects of ALT on apoptosis and autophagy, whereas AP2M1 overexpression inhibited autophagy and induced apoptosis. These findings indicate that AP2M1 serves an important role in the anti-tumor activity of ALT. Some studies have shown that AP2M1 is related to the occurrence and development of cancers, including medulloblastoma, head and neck squamous cell carcinoma, esophageal squamous cell carcinoma, chronic myeloid leukemia, prostate cancer and HCC [8, 19, 20, 29, 30]. Functionally, AP2M1 has been shown to function in clathrin-mediated endocytosis, which regulates communication between cells and their environments. It is well known that endocytosis plays a role in delivery of pharmacologically active substances, such as synthetic drugs, natural compounds, gene material and many other pharmaceutical products [31, 32]. Recent studies show that AP2M1 mediated endocytosis also participates in the internalization of signaling receptors and the regulation of cell signaling. Meisel Sharon et al. [20] showed that AP2M1 serves a mechanistic role in insulin-like growth factor-1 receptor internalization. Mikula et al. [33] reported that AP2M1 is essential for the recruitment of receptor tyrosine kinases and components of signal transduction cascades to chromatin containing genes which are transcribed. In addition, some studies have preliminarily explored the mechanisms of AP2M1. Mélanie Le Duff et al. [17] showed that the downregulation of AP2M1 in cancer cells significantly reduced the sensitivity to soluble signals generated from senescent cells, thereby inhibiting cell invasion. Further, following apoptosis induction, AP2M1 expression varies in different types of cells. In addition, AP2M1 is positively associated with cyclin D1 in adenoid cystic carcinoma (AdCC) and mucoepidermoid carcinoma (MEC), indicating that AP2M1 are involved in the proliferation of AdCC and MEC to cause tumor growth [18]. In the present study, we found that AP2M1 not only regulated the expression of autophagy-related markers, such as Beclin 1, LC3 and p62, but also modulated the expression of apoptosis-related markers bcl-2, bax and cleaved caspase3. These data indicate that AP2M1 acts as an upstream signal molecule regulating cell apoptosis and autophagy. Therefore, based on previous studies and the results of our findings, we speculate that AP2M1 may promote the internalization of certain signalling receptors and inactivation of downstream effectors to regulate cell autophagy and apoptosis and additional well-designed studies will validate these hypothesis of AP2M1’s involvement in ALT mediated anti-tumor activities.

## Conclusion

Taken together, we showed that ALT was able to prevent the proliferation of ALL cells by inducing apoptosis and inhibiting autophagy. The underlying mechanism involved the regulation of AP2M1.

## Data Availability

The datasets used and/or analyzed during the present study are available from the corresponding author on reasonable request.

## Abbreviations

ALL: Acute lymphoblastic leukemia
ALT: Alantolactone
GSK: Glycogen synthase kinase
ER: Endoplasmic reticulum
FBS: Fetal bovine serum
si-AP2M1: siRNA for AP2M1
si-Beclin1: siRNA for Beclin1
FITC: Fluorescein Isothiocyanate
PI: Propidium iodide
MMP: Mitochondrial membrane potential
BCA: Bicinchoninic acid assay
SDS-PAGE: Sodium dodecyl sulfate–polyacrylamide gel electrophoresis
PVDF: Polyvinylidene fluoride
ECL: Enhanced chemiluminescence
OD: Optical densities
DAB: Diaminobenzidine
SD: Standard deviation
